# Automated Mathematical Algorithm for Quantitative Measurement of Strabismus Based on Photographs of Nine Cardinal Gaze Positions

**DOI:** 10.1155/2022/9840494

**Published:** 2022-03-24

**Authors:** Yena Christina Kang, Hee Kyung Yang, Young Jae Kim, Jeong-Min Hwang, Kwang Gi Kim

**Affiliations:** ^1^Department of Biomedical Engineering, Gil Medical Center, Gachon University College of Medicine, Incheon 21565, Republic of Korea; ^2^Department of Ophthalmology, Seoul National University Bundang Hospital, Seoul National University College of Medicine, Seoul 03080, Republic of Korea

## Abstract

This study presents an automated algorithm that measures ocular deviation quantitatively using photographs of the nine cardinal points of gaze by means of deep learning (DL) and image processing techniques. Photographs were collected from patients with strabismus. The images were used as inputs for the DL segmentation models that segmented the sclerae and limbi. Subsequently, the images were registered for the mathematical algorithm. Two-dimensional sclera and limbus were modeled, and the corneal light reflex points of the primary gaze images were determined. Limbus recognition was performed to measure the pixel-wise distance between the corneal reflex point and limbus center. The segmentation models exhibited high performance, with 96.88% dice similarity coefficient (DSC) for the sclera segmentation and 95.71% DSC for the limbus segmentation. The mathematical algorithm was tested on two cranial nerve palsy patients to evaluate its ability to measure and compare ocular deviation in different directions. These results were consistent with the symptoms of such disorders. This algorithm successfully measured the distance of ocular deviation in patients with strabismus. With complementation in the dimension calculations, we expect that this algorithm can be used further in clinical settings to diagnose and measure strabismus at a low cost.

## 1. Introduction

Strabismus is a condition in which the eyes do not line up properly: while one eye points towards a particular object, the other deviates in another direction [1]. Although the cause is still not clearly understood, it is well known that several factors may affect the occurrence of this disorder including age, inheritance, ethnicity, and history of other ocular diseases [2–4]. Strabismus can lead to serious ocular disorders, such as amblyopia, diplopia, and permanent vision loss, if it remains untreated. Therefore, timely and accurate diagnosis is extremely important.

At present, there are several methods for diagnosing and measuring strabismus clinically. The Prism Cover Test (PCT) measures the magnitude of the deviation using a piece of prism [5, 6]. It is the most widely used and easy method for examining strabismus, but it exhibits an interrater reliability problem as the examination is strongly dependent on the examiner experience, examiner bias, and positioning of the prism [5]. Furthermore, as this method requires patient cooperation [7], the PCT is not appropriate for infants, who constitute the majority of the patient population. The Hirschberg test quantifies ocular deviation based on the position of the corneal light reflex point by measuring the distance between the reflex point and limbus center [8]. The Krimsky test uses hand-held prisms to determine the strabismus angle by examining the amount of prism that is required to position the reflex point correctly [8]. Both tests can calculate the extent of the deviation without the cooperation of the subject; however, the results rely on the examiner ability, which suggests lower interrater reliability. Moreover, both tests require a certain correction of the angle kappa which is the difference between the primary line of sight and pupillary axis to avoid the overestimation of the strabismus deviation [9].

Despite the importance of early detection and the acknowledgement of the inaccuracy of existing diagnostic systems, current clinical practice still tends to adhere to these traditional methods as relevant research is lacking. Although various attempts to diagnose strabismus are currently underway, studies regarding the accurate measurement and quantification of the deviation angle to determine the severity of strabismus remain lacking. Chen et al. [10] developed a system that recognizes strabismus using eye-tracking data and a convolutional neural network (CNN). They collected gaze deviation images with their eye-tracking system and applied the CNN to classify the image as positive or negative. Miao et al. [11] integrated image processing with virtual reality to develop an automated device for effective strabismus diagnosis. Weber et al. [12] proposed a pair of portable goggles that can measure strabismus objectively; the goggles implement a modified Hess screen test [13] by measuring the ocular deviations on a nine-point target grid, and the results are compared with actual Hess screen test results. Lim et al. [14] used image processing to measure the ocular deviation angle in different directions of gaze using image editing software.

Despite the exponential development of artificial intelligence in recent years, few studies have demonstrated the use of such techniques. To overcome the difficulties of previous studies, the aim of this study was to propose a mathematical algorithm for measuring strabismus quantitatively using photographs of the nine cardinal gaze positions (nine gazes), by integrating deep learning (DL) to segment the area of the eyes and image analysis to provide automated and accurate strabismus measurement.

## 2. Materials and Methods

### 2.1. Data Collection

**Figure 1:**
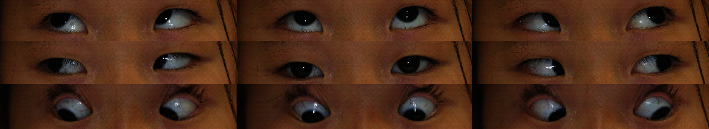
Nine cardinal gaze points.

Photographs were collected from strabismus patients of different ages and genders. Each patient captured nine photographs of his/her upper face while gazing at different points representing the nine gazes (Figure 1). These photographs were obtained with an 8.2-megapixel digital single-lens reflex camera (EOS20D; Canon Inc., Tokyo, Japan) with a ling flash attached on the camera lens. The camera was positioned 1 m from the patient. Moreover, it was ensured that a corneal reflex point was present in each eye of the patient image with a primary gaze, as these points were crucial for the image processing. The resolution of the images was fixed to 2544 × 1696 pixel.

For the DL-based segmentation, 529 images were used for training, 133 were used for validation, and 166 were used for testing the performance of the trained model. The nine gaze images of two adult patients with paralytic strabismus were used to test its performance for the postsegmentation algorithm.

### 2.2. Data Preprocessing

The data preprocessing and postsegmentation processing, which included the mathematical algorithm, were performed on Python (version 3.8.8; Python Software Foundation, Wilmington, DE, USA) and OpenCV library. The collected photographs were manually preprocessed before they were used for the DL training. First, the areas of the limbi and sclerae for both eyes were manually annotated in ImageJ (NIH, Bethesda, MD, USA) for use as the ground truth images. These were created as mask images and used as inputs for the DL training along with the nine gaze images. Furthermore, the images were resized to 512 × 512 pixels, as the inputs of convolutional networks must have pixel size with the same width and height.

### 2.3. Sclera and Limbus Segmentation

**Figure 2:**
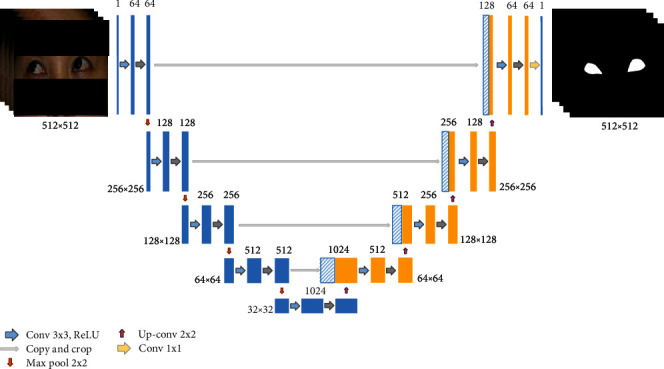
Architecture of U-Net model.

In this study, the U-Net architecture was applied to train the DL model for the limbus and sclera segmentation. U-Net is a CNN that is popular in medical image segmentation [15]. It has a symmetric architecture with an encoder (contracting) path and a decoder (expansive) path (Figure 2). The encoder path (left) consists of two iterations of a 3 × 3 convolution for each convolutional network. Each network is followed by ReLU and batch normalization using a 2 × 2 max pooling operation. Every operation halves the spatial dimension, while doubling the feature channels. The decoder path (right) uses a 2 × 2 up-convolution for up-sampling, which doubles the spatial dimension and halves the feature channels. Thereafter, the corresponding feature map from the encoder is concatenated with the one in the decoder, and subsequently, two iterations of 3 × 3 convolution are applied, followed by ReLU. Finally, a 1 × 1 convolution is applied to map and output the final feature map, which is a mask image of the segmented parts in this case [16, 17].

**Figure 3:**
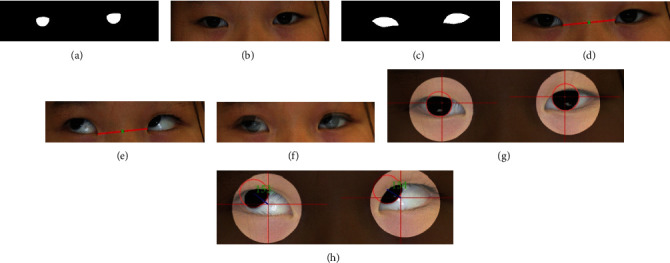
Summary of the overall process of the algorithm. The sample images that are put into the segmentation model: (a) example of ground truth images of limbus, (b) example of raw data, and (c) example of ground truth images of sclera. The registration process: (d) example of center image with reference line (red line) drawn and (e) example of rotating image with reference line (red line) drawn. The center (green dot), length, and angle of the reference line are adjusted to be the same as those of (e) for registration to take place. (f) The overlaid image of (d) and (e), showing that both eyes are positioned on the same location. (g) Example of recognized limbi (red circle) and the corneal reflex points (intersect of the parallel and vertical lines). (h) Example of recognized limbus (red circle) and measured distance between the limbus center and the corneal reflex point detected in (g).

Two separate models were trained for the limbus and sclera segmentation, in which two sets of ground truth images were used for each photograph (Figures 3(a)–3(c)). Both models were trained using the Adam optimizer with a batch size of 4, a learning rate of 0.0001, and 200 epochs.

### 2.4. Mathematical Algorithm

#### 2.4.1. Image Registration

Image registration of nine gaze images was implemented before performing any pixel-based measurements (Figures 3(d)–3(f)). The registration was performed based on the primary gaze image of each patient. This process was necessary because the distance measurement would be meaningless if the eyes of a single patient were not positioned at the same location with the same size.

Several geometrical features were measured for use as indicators of the two-dimensional (2D) transformation. First, the primary gaze image was selected from the nine inputs. Subsequently, using the trained model, the area of the sclerae was segmented using the trained model, and the output mask image was resized to the raw data size. Reference points that represented the innermost points of both sclera areas were then defined using the output image. A line was drawn connecting the reference points, which could be used to determine three elements: the center, length, and angle of the line.

An image with a different gaze was considered, and the previous steps were repeated to obtain the three elements. Thereafter, 2D transformation was performed using the values. First, translation was performed by moving the reference center to the same position with the reference center of the primary gaze image. Subsequently, rotation and scaling were applied to ensure that the length and angle of the reference image were the same with those of the primary gaze image. Figures 3(d)–3(f) present an example image before and after the registration. By adjusting the points of the innermost parts of the sclerae, both eyes were positioned at a similar location pixel-wise and had a similar size.

#### 2.4.2. 2D Limbus Modeling and Definition of Corneal Reflex Point

Several assumptions were made based on the limitations of analyzing three-dimensional objects in a 2D environment. The assumptions were as follows:

1. Both the limbus and sclera are perfect spheres
2. In adults, the radius of the sclera is 2.5 times longer than that of the limbus
3. The extension line of the corneal light reflex point penetrates the center of the eyeball

Assumption 2 was defined based on the average eye size that is used in clinical settings [18, 19]. The radius of the limbus and corneal reflex point of the primary gaze image, which was further used as an indicator of the other images for the measurement, were defined. All of the images were registered prior to processing. The limbus segmentation was performed for the primary gaze image using the trained model, followed by resizing of the output mask image to match its size with the raw data. Using the mask image, the smallest enclosing circles were drawn to recognize each limbus (Figure 3(g)). Thereafter, the radius of the recognized limbus was measured.

The raw images and segmented masks were added to mask the area that was not the limbus for the corneal reflex point. Subsequently, the area of the bright point was determined. The exact coordinates of the reflex point were the center of the bright point area that was determined.

#### 2.4.3. 2D Sclera Modeling

The sclera of the eye was also modeled with the consideration of a follow-up study as well as to provide improved visualization. The initial steps were the same as those of the limbus modeling, namely, registering the image and segmenting the sclerae area. Subsequently, enclosing circles were drawn to determine the center point of each sclera. Finally, a 2D sclera model was created by drawing a circle with a radius that was 2.5 times longer than that of the limbus, based on the assumption stated in the previous section.

#### 2.4.4. Limbus Recognition and Distance Measurement

The final step of the algorithm was to recognize limbi from the nonprimary gaze images and to calculate the pixel-wise distance between the limbus center and the corneal reflex point that was obtained from the primary gaze image. To achieve this, a fitting ellipse was drawn on the segmented limbus. Thereafter, a line representing the short axis was drawn. The limbus center was determined by assigning it to the extension line of the short axis with the length of the limbus radius, starting from the outer contour of the ellipse. A circle was drawn with the newly assigned center and radius of the limbus, thereby finalizing the limbus recognition process (Figure 3(h)). The distance between the limbus center and corneal reflex point was calculated.

### 2.5. Evaluation Method

A confusion matrix was generated to evaluate each segmentation model. The confusion matrix used various evaluation indices, such as the accuracy, sensitivity, specificity, and dice similarity coefficient (DSC), to assess the performance of the model.

For the algorithm, the line and measured distance were displayed on the raw images to confirm that the limbus recognition and distance measurement were performed properly, as well as to provide improved visualization. Moreover, we considered a couple of paralytic strabismus patients to measure the differences in movement between the two eyes. We could obtain examples of two cases: fourth cranial nerve palsy and sixth cranial nerve palsy. Fourth cranial nerve palsy deteriorates eye movement, preventing downward and inward movement [20], whereas sixth cranial nerve palsy causes esotropia impairing outward eye movement [21]. Thus, it is necessary to observe movements of the limbi based on various directions of gaze.

## 3. Results

### 3.1. Segmentation Models

The segmentation models for the sclera and limbus were tested using 166 images. Confusion matrices were used for the evaluation method, which were created by comparing the ground truth images and the resulting images pixel by pixel. The comparisons were represented using true positive (TP), true negative (TN), false positive (FP), and false negative (FN). The accuracy, sensitivity, specificity, and DSC were calculated with these values using Equations (1)–(4). The results are presented in Table 1.

(1) $$\begin{array}{c} \text{Accuracy}=\frac{\text{TP}+\text{TN}}{\text{TP}+\text{TN}+\text{FP}+\text{FN}}\times 100, \end{array}$$

(2) $$\begin{array}{c} \text{Sensitivity}=\frac{\text{TP}}{\text{TP}+\text{FN}}\times 100, \end{array}$$

(3) $$\begin{array}{c} \text{Specificity}=\frac{\text{TN}}{\text{FP}+\text{TN}}\times 100, \end{array}$$

(4) $$\begin{array}{c} \text{DSC}=2\times \frac{\text{TP}}{\left(\text{TP}+\text{TN}\right)+\left(\text{FP}+\text{FN}\right)}. \end{array}$$

According to Table 1, the sclera segmentation model exhibited an average accuracy of 99.84%, sensitivity of 97.47%, specificity of 99.90%, and DSC of 96.88%. Similarly, the limbus segmentation model also provided high values: accuracy of 99.92%, sensitivity of 95.63%, specificity of 99.96%, and DSC of 95.71%. These results demonstrated that the trained models could correctly segment the limbi and sclerae of the eyes on the image, which means that the images that were segmented by these models could be used as the inputs for the subsequent algorithm.

### 3.2. Algorithm

The algorithm was evaluated using visualization and testing of specific cases. Figure 3(h) depicts the manner in which the visualization was performed; it illustrates which part of the image the algorithm attempted to measure and displays the actual number so that the results can be understood more clearly.

As it is difficult to estimate the actual amount of movement using a photograph and the pixel-wise distance does not represent any clinically meaningful indicators, we used the ratio of the impaired eye movement to normal eye movement to test the performance of the algorithm. Two specific cases of strabismus that required examination with the nine gazes were considered: fourth cranial nerve palsy and sixth cranial nerve palsy.

**Figure 4:**
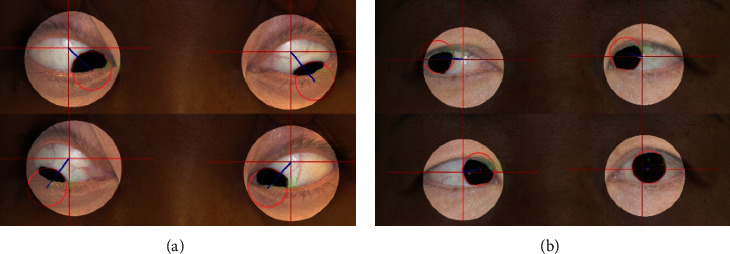
(a) Algorithm result for patient 1. The pixel-wise distances that are measured indicate that the patient has difficulties moving eyes inwards. (b) Algorithm result for patient 2. The pixel-wise distances that are measured indicate that the patient has difficulties moving left eye outwards.

We attempted to observe the deviation by measuring the ocular movement of two patients with cranial nerve palsy (Table 2). The first patient was diagnosed with fourth cranial nerve palsy in both eyes. We observed that her eyes performed less movement when trying to move inwards and downwards, as the percentage of movement was 91.6% for the left eye and 89.4% for the right eye. The second patient had sixth cranial nerve palsy in her left eye, and it was clearly demonstrated that the ability of the left eye to move outwards was damaged as its movement was only 27.6% compared to the right eye, whereas inward movement remained similar.  Figure 4 clearly visualizes the ocular deviation of two separate cases, depicting how the measurements were taken place.  Figure 4(a) represents the result of patient 1, whereas Figure 4(b) represents the result of patient 2.

## 4. Discussion

The purpose of this study was to introduce a mathematical algorithm to measure the ocular deviation in strabismus patients using photographs of the nine cardinal gaze positions. The overall procedure consisted of two steps: segmentation of the sclera and limbus using U-Net architecture, and distance measurement between the limbus center and corneal reflex point using an image processing technique. The segmentation models yielded significantly accurate results, which means that the trained models could accurately segment the sclera and limbus areas so that they could be used for further image processing steps, whereas the mathematical algorithm exhibited a clear distance measurement and meaningful explanations for the disorders that were present in the testing images. The results suggest that the algorithm offers the potential to be applied to actual clinical settings with several modifications that would make it more useful and convenient.

Several attempts have been made to study ocular movements using images of different gaze positions [22–26]. Figueiredo et al. [23] developed a web application using a CNN to classify eye versions into the nine gaze positions, but it was not clear how this could contribute to the actual examination of strabismus. Zheng et al. [26] implemented a DL algorithm to classify horizontal strabismus using primary gaze photographs of children; however, this study lacked other types of strabismus and different gaze positions. To overcome these difficulties, we attempted to build a powerful CNN model to segment the limbus and sclera areas as accurate as possible as it is crucial for objective measurement of strabismus. Furthermore, we combined the developed algorithm and the concept of nine cardinal points of gaze to enable the measurement of ocular deviation from various directions. We expect that this algorithm could aid doctors in the clinical settings to diagnose and classify different types of strabismus and their severities using captured photographs and potentially could further be used for studying different areas of strabismus.

Several limitations of this study necessitate improvements to the algorithm and further research. First, this study analyzed eyes in the 2D plane, whereas real eyes are three-dimensional (3D). Therefore, errors were inevitable when measuring the distance. Although a previous study presented software that quantitatively analyzes binocular misalignment in a 3D environment, a portion of the process remains manual and only primary gaze sources are used [27]. Thus, follow-up study would require this algorithm to be implemented and changed for its application to a 3D model of the eyes. Furthermore, it was difficult to identify the location of the limbi in cases of small eyes with little exposure to the limbus area. To resolve this problem, one suggestion is for the examiners to adjust the location of the limbus manually after approximately determining the location, making the algorithm semiautomatic.

Another problem of automatic and objective measurement is that it does consider the potential for the existence of differences according to the age or surgery status of the patient [28, 29]. An age-related gaze impairment in horizontal and upward gazes has been reported [28]. Therefore, it is necessary for examiners to determine the ocular deviation of subjects considering their age and surgery status, even when using objective measurement methods.

## 5. Conclusion

To conclude, this study has provided an algorithm that can automatically quantify ocular deviation using nine gaze photographs, by combining DL and 2D image processing techniques. The algorithm enables examiners to measure the pixel-wise distance between the limbus center in various gaze positions and the corneal reflex point of the primary gaze image. The proposed method offers the potential to aid ophthalmologists in measuring ocular deviation in strabismus patients with more objective indicators. A follow-up study would require the implementation of the algorithm in an actual web or mobile application or software with an easy and simple user interface to provide 3D application of the analysis.

## Figures and Tables

**Table 1:** Results of segmentation models.

	Accuracy (%)	Sensitivity (%)	Specificity (%)	DSC (%)
Sclera segmentation	99.84	97.47	99.90	96.88
Limbus segmentation	99.92	95.63	99.96	95.71

**Table 2:** Results of algorithm testing images. Patient 1 represents a patient with fourth cranial nerve palsy, in which both eyes exhibit disability with downward and inward movement. Patient 2 represents a patient with sixth cranial nerve palsy, in which the left eye cannot move outwards.

Patient 1				Patient 2			
	Downward, outward (pixels)	Downward, inward (pixels)	Percentage (%)		Left eye (pixels)	Right eye (pixels)	Percentage (%)
Left eye	227	208	91.6	Outwards	45	163	27.6
Right eye	218	195	89.4	Inwards	109	105	103.8

## Data Availability

The datasets generated or analyzed during the current study are available from the corresponding authors upon reasonable request.
